# Travers vs. Boardman: An Action of Tort, and What It Teaches

**Published:** 1881-03

**Authors:** 


### Selections.

### TRAVERS vs. BOARDMAN: AN ACTION OF TORT, AND WHAT IT TEACHES.

THE following excellent editorial, in a recent number of the *Boston Medical and Surgical Journal*, seems to have more than a local interest, and considering the importance of the subject, we give the article in full.

"An action for damages was tried last week before the superior court of this county, wherein the defendant was a well-known and reputable physician of Boston. The medical elements involved in the suit are so peculiar that we believe a summary of the case will be instructive to our readers. The material facts brought to light at the trial were as follows: In the autumn of 1878, a woman, the plaintiff in this case, applied at the Boston City Hospital for treatment for some uterine disease; she was referred to Dr. W. E. Boardman, the regular physician on duty in the department for such patients. In the course of the treatment, which extended through more than two months, Dr. Boardman, in the presence and with the assistance of Dr. Wyman, the medical externe, performed on one occasion the simple operation of puncturing the cervix for local depletion, the knife being used, of course, with a vaginal speculum in position. This was the only operation to which the patient was ever subjected at Dr. Boardman's hands. The case was dismissed as convalescent before Christmas; and at that time the woman testified her appreciation of the services rendered by urging Dr. Boardman to accept some tokens of her regard. The next that was heard of the case was about three weeks later, when the patient re-appeared, charging Dr. Boardman with malpractice, and insisting that he should 'do something for her.' This was a premonitory symptom of her present 'complaint,' which, as presented by her, under oath, in court, before a crowd of men, is in effect as follows: She alleges that at her fourth and last visit to the hospital, Dr. Boardman, with Dr. Wyman's assistance, placed her in position on a table, and then, without her permission, and, as she believes, for experimental purposes only, amputated her clitoris, and with it excised a piece of the mucous membrane of her vulva 'as large as her hand;' that she bled freely after this operation; that she knows the parts removed to have been as she describes, for she saw them in a basin where the doctors placed them; that as soon as the operation was finished she arose from the table and went home; that in consequence of that operation she has been in mental and physical misery most of the time in the interval since; her menstruation has stopped; she has spasmodic pains; she has lost many pounds in weight; she is disabled for her ordinary work; she has distressing hallucinations; and she has been obliged to break an engagement of marriage, because, with the loss of her clitoris, she has found on trial that her sexual desire has departed; that she knows her clitoris is gone, for she has examined her person with the aid of a mirror. For this loss, and for its direct and indirect consequences, she claims damages from Dr. Boardman to the amount of $3,000.00. Her allegations as to the mutilation of her vulva were supported at the trial by the testimony of one of her neighbors, a spinster, who examined the Travers' genitals soon after the alleged operation, and found 'a piece of the clitoris gone;' also by the testimony of two men (who are unknown to fame or to the regular profession, but who, because they write "Dr." before their names are by that mark as good as the best in a court of law), one of whom swore that he was of the opinion that the clitoris had beed amputated, for he had examined the woman more than a year after the alleged malpractice, and had observed a linear scar at the place where the clitoris ought to be. The cross-examination of these foreign gentlemen was extremely racy. The only other inspection to which, so far as the testimony showed, the woman had submitted herself, was on one occasion when Dr. J. A. Lamson, a regular physician, examined her in company with another well-known practitioner; both agreed that they found no defect or loss of tissue whatever about the genitals, the spot indicated by the plaintiff as the scar of the operation being the meatus urinarius, and readily admitting a female catheter. Such are the essential facts of this case.

"We regret that we have not the space for an extended abstract of the Honorable Judge Putnam's charge to the jury. It contained but few morsels of encouragement for the audacious plaintiff. He said distinctly that for the jury the question was one mainly of veracity; if the jury had any doubt of the woman's story, for upon her rested the burden of proof, then they must bring a verdict for the defendant. To assist the jury to understand the full bearings of the case, his honor stated very clearly the principles of law involved; that in a physician's dealings with his patient there is an implied contract, as distinguished from a written or expressed contract; that in discharging this contract, a physician must possess a reasonable degree of that skill, learning, and experience ordinarily possessed by physicians living at the time; that in the exercise of this skill, learning, and experience, he needs to use ordinary care; and that as regards liability, it makes no difference whether or not the service rendered was gratuitous.

"The jury deliberated many hours on the simple question of veracity before them, and at length reported that they were unable to agree; it was stated on good authority that they were equally divided.

"Now, this case suggests many reflections; we can mention two or three only of the comments that occur to us. In the first place, this trial illustrates the deplorable facility with which such actions may be brought, and the outrageous advantage which designing persons have in bedaubing honorable physicians with the slime of innuendo and suspicion. The nastiest strumpet in the town has it in her power thus to annoy the best and noblest man among us. In dispensary and hospital practice, as well as in the privacy of office work, the physician of highest skill and purest motives may fall afoul of one of these harpies, may unsuspectingly do the simplest operation for her relief, and a few months later may find himself in court, a defendant in a suit wherein he is accused of doing things which he never did or dreamed of doing, the doing of which, indeed, would stamp him as a fool or a knave. Until some right of supervision is exercised by judges so that such fraudulent suits are prevented from obtaining a place on the docket, or until the law requires that all costs, including the defendant's counsel fees, shall be paid by the plaintiff if he loses his cause, these miserable actions of tort will be springing up, smirching the reputations of honorable men, and displaying their names in the daily press in such a manner as to mislead the public into a feeling akin to a belief that the accusation is more than half true.

"Then what shall we say of the 'trial by jury?' In this case six men believed the utterly improbable story of this female Thersites, who babbled her filthy yarn with indecent freedom, and was able, by her vile allegations, to neutralize in their minds all that could be brought to contradict her or to show the inherent improbability of her nasty narrative. Such a phenomenon is not reassuring to honest suitors, and does not inspire reverence, or this ancient method of administering justice.

"Of the conduct and character of so-called professional men, whether lawyers or physicians, who lend themselves to promote the prosecution of such actions at law as the present, of their motives, their behavior in court, their expectation of reward, we forbear to make extended criticism; we have an opinion which if expressed might not be considered complimentary to those privy counselors.

"Finally, the present case is an illustration, for the thousandth time, of the need of a change in the methods of using medical experts. If, before the trial, any one of the able physicians there present had been agreed upon by both parties, or, in default of such agreement, had been appointed by the court, to present and interpret the medical data of the case, including proper notes of the plaintiff's present physical and mental condition, the sneer of her counsel that 'the doctors all hang together, of course they do; that's to be expected,' would not have been heard; indeed, the case would probably have been taken from the jury. When the choice and testimony of medical experts in civil and criminal suits rests by authority on some more substantial foundation than mere partisan expediency and availability, we shall not be obliged to read in the newspapers, between the lines of the court calender, that the jury in a given case were unable to agree, because half of them considered a scheming woman and two irregular practitioners more trustworthy than six of the best-known among the regular physicians of Boston.

"We hope that Dr. Boardman will insist on a new trial before another jury, for the matter ought not to rest in its present undecided state. He is sure of the sympathetic support of the administration and medical staff of the City Hospital; indeed, he may regard the entire profession as his allies, in a matter touching so intimately the common interest."

